# Analysis of the Elemental Composition of Milk Formulae: Impact on the Nutritional Status of Infants From Birth to 1 Year of Age

**DOI:** 10.1007/s12011-025-04687-x

**Published:** 2025-06-17

**Authors:** Małgorzata Dobrzyńska, Zofia Wojciechowska, Karol Jakubowski, Hanna Markowska, Juliusz Przysławski, Przemysław Niedzielski, Sławomira Drzymała-Czyż

**Affiliations:** 1 Department of Bromatology, Poznan University of Medical Sciences, Rokietnicka 3 Street, 60-806 Poznan, Poland; 2 Department of Analytical Chemistry, Faculty of Chemistry, Adam Mickiewicz University, Uniwersytetu Poznanskiego 8 Street, 61-614 Poznan, Poland

**Keywords:** Infant nutrition, Infant formula, Follow-on formula, Formula for special medical purposes, Elements, Toxic elements

## Abstract

Inappropriate concentrations of elements in infant formulae may lead to adverse health effects, so this study was designed to determine the concentration of selected basic (Na, K, Ca, P, Mg, Fe, Zn, Cu, and Mn) and toxic or potentially toxic elements (As, Cd, Co, Cr, Hg, Sn, and Ni) in formulae for infants from birth to 12 months currently available on the Polish market. The concentrations of these elements were assessed using inductively coupled plasma mass spectrometry (ICP-MS), and the estimated daily intake (EDI) of elements was calculated. The concentrations of most analyzed elements in formulae were in good agreement with the recommended EU limits, except for Cu (median 72 µg/100 kcal, range 0 − 338 µg/100 kcal) and K (155 µg/100 kcal, 103 − 316 µg/100 kcal) in some formulae did not meet the guidelines for minimum and maximum levels. Additionally, there were large differences in Mn concentrations between the various formulae (range 1 − 91 µg/100 kcal). The Ni EDI exceeded 4.3 µg in some formulae, which EFSA established as the lowest observed adverse effect level (LOAEL) for eczematous skin reactions. Therefore, regular monitoring of the composition of formulae for infants is recommended.

## Introduction

During infancy, breast milk is the main source of nutrients for infants, providing an adequate supply of nutrients that support growth, development, and health. The World Health Organization (WHO) recommends exclusive breastfeeding for the first six months of life, but this is not always possible, so milk formulae is an appropriate alternative. Their composition is similar to breast milk and the content of basic nutrients, including minerals, is strictly defined by the Scientific Committee on Food (SCF), the European Food Safety Authority (EFSA), and the European Union Directive guidelines [1, 2].

Globally, less than one in two (around 40–48%) infants ages 0–5 months are exclusively breastfed [3–6]. In Poland, despite a significant increase from 1988, the percentage of infants breastfed is low, with 40% of infants exclusively breastfed until 3 months of age, while 8% are breastfed until 6 months [7]. This means that formulae play a key role in infant nutrition, so ensuring the highest quality is imperative.

Currently, there are few studies that examine the concentration of elements in infant formulae by analyzing a large number of different formulae [8–10]. Most studies analyze only a small number of samples, comparing different types of formulae—such as cow’s, soy, goat’s, or hypoallergenic formulae—or assessing only selected elements. One of the significant studies was conducted by Pandelova et al. This study analyzed only 10 elements (Ca, Cd, Cu, Fe, Hg, Mn, Ni, Pb, Se, and Zn) in the most commonly consumed infant formulae in Europe. The results showed that the levels of analyzed elements in soy-based infant formulae were higher than those found in cow’s milk-based formulae. In some of the analyzed samples, elevated Hg levels of 113 μg/kg were found [8]. Chajduk et al. analyzed essential and toxic elements (As, Cd, Co, Cr, Cu, Fe, Mn, Mo, Ni, Pb, Se, V, and Zn) in just six infant formulae available on the Polish market. The concentrations of essential elements were consistent with the values supplied by the manufacturers, and the concentrations of toxic elements were low [11].

Purkiewicz et al.’s study analyzed the concentrations of selected elements (Ca, Mg, Na, K, P, Fe, Zn, Cu, and Mn) in eight conventional and six formulae for special medical purposes available on the Polish market. In this study, concentrations of Na, Zn, Cu, and K in some of the analyzed samples (formulae for special medical purposes) deviated from the nutritional standards established by the European Commission, especially Cu concentrations, which exceeded the standards by up to 40% [12]. Jardí Piňana et al. determined the concentrations of Na, Mg, P, K, and Ca in 31 infant formulae available on the Spanish market. The concentrations in the analyzed formulas were in line with EU guidelines [13]. Papachristodoulou et al. assessed K, Ca, Fe, Zn, Br, Rb, and Sr concentrations in 28 infant formulae widely distributed in the Greek market. The concentrations were within the limits recommended by the European legislation, with only a few exceptions [14]. There is still a lack of comprehensive studies in the available literature assessing the elemental composition of milk formula in a broad perspective—covering all commercially available milk formulas and analyzing a wide range of elements.

Taking into account the above arguments and the awareness of the crucial role proper nutrition plays in infant development—as well as the negative effects of both nutrient deficiencies and excesses—the concentration of selected elements was determined in milk formulae available on the Polish market for infants up to 12 months of age.

## Materials and Methods

### Materials

All available powdered milk formulae in the Polish market from 2019 to 2023 (*n* = 149) designed for consumption during the first year of life were analyzed, including 37 infant formulae, 34 follow-on formulae, and 78 formulae for special medical purposes. The formulae were analyzed based on cow milk proteins (*n* = 71), goat milk proteins (*n* = 15), soy (*n* = 3), extensively hydrolyzed (*n* = 26), amino acid–based (*n* = 6), preterm (*n* = 4), anti-reflux (*n* = 7), lactose-free (*n* = 5), and comfort formulae (*n* = 12). Samples were coded and stored in conditions complying with the manufacturer’s requirements. The certified reference material, skimmed milk powder ERM (BD150; European Reference Materials, Belgium) was used to validate the analytical methods. Depending on the formula, one, two, or three parallel samples were tested (four milk samples in two repetitions, twenty milk samples in three repetitions, and the rest in a single repetition). The number of repetitions depended on both the availability of the formula and the cost of the study.

### Sample Preparation and Element Composition

The concentrations of basic elements (Na, K, Ca, P, Mg, Fe, Zn, Cu, and Mn) and toxic or potential toxic elements (As, Cd, Co, Cr, Hg, Sn, and Ni) were determined in the Department of Analytical Chemistry, Faculty of Chemistry, Adam Mickiewicz University using inductively coupled plasma mass spectrometry (ICP-MS). All analyses were performed in triplicate and were based on the method described by Yaman et al. [15]. Sample digestion was performed using a microwave accelerated reaction system (MARS6, CEM Corporation, Matthews, NC, USA). Each sample (0.5 g) was weighed into a microwave vessel and filled with 10 mL of 69% ultra-pure nitric acid (ROMIL, Cambridge, UK). The vessels were gently mixed and left open for 15 min to allow sample pre-digestion before microwaving: ramping from ambient temperature to 180 °C over 20 min and then holding the temperature for 20 min. After cooling, the solutions were transferred to 15-mL volumetric flasks and diluted to 15 mL with ultrapure water. Simultaneous analysis of the reference material validated the methods. Nitric acid 65%, Suprapur® was used for preparing standards. Commercial standards (Romil, UK) Na, K, Ca, P, Mg, Fe, Zn, Cu, Mn, As, Cd, Co, Cr, Hg, Sn, and Ni were used for calibration. High-pure gases (N–5.0, purity 99.999%), i.e., argon, helium, and hydrogen (Linde, Poland), were used during ICP MS analysis. All samples were diluted in ultra-pure water with a resistivity ≥ 18 MΩ cm (Milli-Q system, Merck Millipore, Germany).

During ICP MS analysis, the repetition sample was measured several times. Based on this, the precision of the analytical process determined as RSD was less than 20%. Then, 20% is the propagated uncertainty for the whole analytical procedure. This value accounts for relevant sources of variability, including sample inhomogeneity.

Limit of detection (µg/L) is presented as follows: As: 6.0, Ca: 250, Cd: 0.45, Co: 1.5, Cr: 0.77, Cu: 2.4, Fe: 61, Hg: 18, K: 140, Mg: 150, Mn: 1.2, Na: 110, Ni: 13, P: 3100, Zn: 5.6, and Sn: 0.27.

To ensure quality control of the analysis, certified reference materials (CRMs) were used. While the article specifically mentions ERM-BD150 (skimmed milk), additional CRMs were applied for broader validation of the analytical method, and acceptable recovery (80–120%) was obtained for most elements.

### Data Analyses

The element concentrations were compared to the compositional criteria regarding the minimum and maximum content in formulae for infants specified by the European Union guidelines laid down in Commission Directive 2016/127 of September 25, 2015, supplementing Regulation (EU) No 609/2013 of the European Parliament and of the Council regarding the specific compositional and information requirements for infant formula and follow-on formula [16] and Commission Delegated Regulation (EU) 2016/128 of September 25, 2015, supplementing Regulation (EU) No 609/2013 of the European Parliament and of the Council as regards the specific compositional and information requirements for food for special medical purposes [17].

The data were converted to mg or µg per 100 kcal formulae and divided into three groups: infant formulae, follow-on formulae, and formulae for special medical purposes. The estimated daily intake (EDI) of the elements was calculated for all formulae. For formulae intended from birth to 6 months (infant formulae), the EDI was calculated for infants aged 1, 3, and 6 months. For formulae intended for infants aged 7 to 12 months (follow-on formulae), the EDI was calculated for infants aged 7 and 10 months. The formulae for special medical purposes were further divided into three groups depending on the age range of infants for whom they were intended. In formulae intended for infants from birth to 6 months, the EDI is the same as in infant formulae calculated for infants aged 1, 3, and 6 months. For formulae intended for infants aged 7 to 12 months, the EDI is the same as in follow-on formulae aged 7 and 10 months. For formulae intended for infants from birth to 12 months, the EDI was calculated at 1, 6, 7, and 10 months.

Recommendations on energy intake values were used to estimate the EDI of selected elements based on current data from the Stan et al. study, while average body weights were considered based on the current WHO percentiles for infants [18].

It is of note that energy demand in infants after 6 months is not fully covered by energy from formulae (complementary foods are introduced); therefore, it was assumed that infants in the 7 th month of life obtain 45% of energy from formulae and 25% in the 10 th month [19].

The results were compared with the nutritional guidelines applicable in Poland as per the EFSA. The EDI was compared to the adequate intake (AI) for most elements. The recommended daily intake (RDA) was adopted for Fe and Zn in milk intended for infants after 6 months, with Cu and Zn values compared to the tolerable upper level (UL). Since these values were not developed for infants, data for children aged 1 to 3 years were used, and the values were assumed to be 1 mg/day and 7 mg/day for Cu and Zn, respectively. As, Cd, Hg, and Sn concentrations were compared to maximum levels according to the commission regulations (2023/915 of April 25, 2023) [20]. Ni concentrations were compared to the TDI and the lowest observed adverse effect level (LOAEL) for eczematous flare-up reactions [21]. Cr concentrations were compared to AI according to EFSA recommendations [22].

Minimum and maximum values were calculated for all analyzed formulae, median, and quartiles (first and third). Statistical analyses were performed using Statistica 13.3 (StatSoft, TIBCO Software Inc., Palo Alto, California, USA).

## Results

### Trace Element Concentrations

The concentrations of basic elements in formulae intended for infant feeding (mg/100 kcal or µg/100 kcal) are presented in Tables 1, 2, and 3 and were compared to EU regulations regarding the minimum and maximum levels of minerals in foods intended for infants. The element concentrations in formulae for infants from birth to 6 months of age (infant formulae) in Table 1 show that the concentrations of P and Mn were within the acceptable limits for all formulae (*n* = 37), but Na concentrations were below the minimum level in two formulae (11–17% lower), K concentrations exceeded the maximum level in 16 samples, by an average of 13% (minimum 1%, maximum 32%). Ca concentrations were below the minimum level in one sample (on average 1%), and Mg concentrations exceeded the maximum level in two samples, by 4–6%. Fe concentrations exceeded the maximum level in two samples by 23–69%. Zn was below the minimum level in one sample (20% lower) and exceeded the maximum level in one sample by 27.5%. Cu concentrations were inappropriate in 21 infant formulae, below the minimum level in 16 samples (5–90% lower, and in one sample, below the detection level) and exceeding the EU limit in 5 samples.

**Table 1:** Elements (mg/100 kcal) in analyzed infant formula samples

**Analyzed elements**	Norms*	Infant formulae (*n*= 37) Median (1st–3rd) < min–max >
Na (mg/100 kcal)	25–60	37 (31–41) < 21–56 >
K (mg/100 kcal)	80–160	158 (138–175) < 111–211 >
Ca (mg/100 kcal)	50–140	85 (73–104) < 49–115 >
P (mg/100 kcal)	25–90	61 (49–70) < 40–86 >
Mg (mg/100 kcal)	5–15	12 (9–13) < 6–16 >
Fe (mg/100 kcal)	0.3–1.3	0.6 (0.5–0.8) < 0.3–2.2 >
Zn (mg/100 kcal)	0.5–1.0	0.7 (0.6–0.8) < 0.4–1.3 >
Cu (µg/100 kcal)	60–100	66 (45–81) < 0–280 >
Mn (µg/100 kcal)	1–100	9 (6–21) < 4–46 >

*Appropriate composition of elements in infant formulae as specified by the European Union guidelines

**Table 2:** Elements (mg/100 kcal) in analyzed follow-on formulae samples

**Analyzed elements**	Norms*	Follow-on formulae (*n*=34) Median (1st–3rd) < min–max >
Na (mg/100 kcal)	25–60	38 (31–41) < 23–61 >
K (mg/100 kcal)	60–160	158 (136–172) < 103–289 >
Ca (mg/100 kcal)	50–140	117 (102–129) < 63–179 >
P (mg/100 kcal)	25–90	77 (68–84) < 48–133 >
Mg (mg/100 kcal)	5–15	13 (9–15) < 6–41 >
Fe (mg/100 kcal)	0.6–2.0	1.0 (0.8–1.2) < 0.3–1.8 >
Zn (mg/100 kcal)	0.5–1.0	0.7 (0.7–1.0) < 0.6–1.4 >
Cu (µg/100 kcal)	60–100	81 (52–99) < 3–338 >
Mn (µg/100 kcal)	1–100	17 (9–32) < 2–55 >

*Appropriate composition of elements in follow-on formulae as specified by the European Union guidelines

**Table 3:** Elements (mg/100 kcal) in analyzed formula for special medical purposes intended for infants

**Analyzed elements**	Norms*	**Extensively hydrolyzed formulae** (*n*=26)	**Amino acid-based formulae** (*n*=6)	**Formulae manufactured from goats’ milk proteins** (*n*=15)	**Comfort-formulae** **(*n* = 12)**	**Anti-reflux formulae** **(*n* = 7)**	**Lactose-free formulae** **(*n* = 5)**	**Preterm formulae** **(*n* = 4)**	**Soya-based formula for infants** **(*n* = 3)**	**Total formula for special medical purposes intended for infants** **(*n* = 78)**
		Median (1st–3rd) <min–max>								
Na (mg/100 kcal)	25–60	37 (32–42)	51 (45–54)	36 (30–43)	37 (33–43)	42 (32–49)	38 (33–46)	50 (33–82)	33 (30–50)	38 (32–46)
		< 23–62 >	< 44–59 >	< 24–75 >	< 29–54 >	< 30–55 >	< 25–47 >	< 29–103 >	< 30–50 >	< 23–103 >
K (mg/100 kcal)	80–160	152 (138–175)	160 (137–169)	167 (130–191)	139 (128–151)	161 (134–173)	153 (146–163)	141 (124–164)	133 (129–158)	149 (136–175)
		< 124–207 >	< 113–196 >	< 110–316 >	< 119–183 >	< 132–177 >	< 123–195 >	< 112–182 >	< 129–158 >	< 110–316 >
Ca (mg/100 kcal)	50–250	92 (81–133)	113 (107–118)	100 (82–113)	93 (81–100)	121 (102–141)	117 (86–121)	141 (115–169)	94 (92–96)	101 (86–121)
		< 64–166 >	< 100–134 >	< 54–182 >	< 59–127 >	< 92–148 >	< 69–122 >	< 101–185 >	< 92–96 >	< 54–185 >
P (mg/100 kcal)	25–100	60 (47–80)	77 (60–89)	76 (64–83)	63 (48–68)	68 (58–96)	78 (74–83)	83 (74–91)	102 (65–107)	69 (57–83)
		< 37–93 >	< 54–98 >	< 52–139 >	< 41–83 >	< 58–98 >	< 58–100 >	< 69–95 >	< 65–107 >	< 37–139 >
Mg (mg/100 kcal)	5–15	10 (9–13)	16 (13–17)	12 (9–13)	11 (10–13)	13 (11–14)	12 (10–13)	13 (11–14)	12 (12–13)	12 (9–13)
		< 7–17 >	< 10–17 >	< 7–23 >	< 9–16 >	< 10–16 >	< 8–14 >	< 10–14 >	< 12–13 >	< 7–23 >
Fe (mg/100 kcal)	0.3–2.5	0.9 (0.6–1.1)	1.3 (1.2–1.5)	0.8 (0.7–1.1)	0.8 (0.7–0.9)	0.9 (0.5–1.1)	0.8 (0.7–0.9)	1.3 (1.0–1.5)	0.8 (0.6–0.9)	0.8 (0.7–1.1)
		< 0.4–1.4 >	< 0.5–1.7 >	< 0.4–1.6 >	< 0.6–1.1 >	< 0.4–1.3 >	< 0.7–1.1 >	< 0.8–1.6 >	< 0.6–0.9 >	< 0.4–1.7 >
Zn (mg/100 kcal)	0.5–2.4	0.8 (0.7–0.9)	0.9 (0.9–1.1)	0.7 (0.7–0.8)	0.9 (0.8–1.0)	0.9 (0.8–1.1)	0.9 (0.8–0.9)	1.2 (1.0–1.3)	0.8 (0.8–1.1)	0.8 (0.8–1.0)
		< 0.6–1.1 >	< 0.8–1.1 >	< 0.6–1.4 >	< 0.6–1.4 >	< 0.6–1.2 >	< 0.6–1.0 >	< 1.0–1.3 >	< 0.8–1.1 >	< 0.6–1.4 >
Cu (µg/100 kcal)	60–120	79 (56–89)	87 (54–139)	58 (41–69)	66 (50–88)	78 (64–115)	61 (51–82)	71 (63–78)	66 (43–75)	68 (52–88)
		< 41–136 >	< 52–159 >	< 0–186 >	< 43–177 >	< 53–153 >	< 38–175 >	< 61–81 >	< 43–75 >	< 0–186 >
Mn (µg/100 kcal)	1–100	12 (9–27)	32 (19–42)	19 (10–24)	21 (12–62)	25 (13–69)	24 (23–28)	12 (9–16)	55 (45–63)	19 (11–34)
		< 3–78 >	< 5–66 >	< 1–46 >	< 3–91 >	< 4–76 >	< 9–77 >	< 8–17 >	< 45–63 >	< 1–91 >

*Appropriate composition of elements in formula for special medical purposes intended for infants as specified by the European Union guidelines

Regarding follow-on formulae for infants after 6 months to 12 months (Table 2), Mn concentrations were within the acceptable limit for all samples (*n* = 34). Na concentrations were below the minimum level in two formulae and exceeded the maximum level in two formulae by 2%. K concentrations exceeded the maximum level in fourteen samples, with only one formula exceeding the maximum Ca level (28%). P concentrations exceeded the maximum level in seven samples by an average of 15% (minimum 1%, maximum 48%). Mg concentrations exceeded the maximum level in eight samples by a minimum of 1% and a maximum of 172%. Fe concentrations in one sample were below the minimum level (50% lower). Zn concentrations exceeded the maximum level in four formulae (7–39%). The Cu concentration was below the minimum limit (80% lower) in one sample, whereas seven samples exceeded the maximum limit range by 10–238%.

Table 3 shows the element concentrations of formulae for special medical purposes intended for infants which were further classified into extensively hydrolyzed formulae, amino acid–based formulae, formula manufactured from goat milk proteins, comfort formulae, anti-reflux formulae, lactose-free formulae, preterm formulae, and soya-based formula. The Ca, Fe, Zn, and Mn concentrations were within the acceptable limits for all formulae (*n* = 78). Na concentrations exceeded the maximum level in extensively hydrolyzed formulae (1 sample by 3%), goat milk proteins formulae (1 sample by 20%), and preterm formulae (2 samples 3–72%). Na concentrations in the extensively hydrolyzed formula and goat formula were below the minimum level whereas K concentrations exceeded the maximum level in 33 samples, regardless of the formulae type (minimum 1%, maximum 98%) the except for the soya-based formula which met the limits. P concentrations exceeded the maximum level in one sample of goat milk formula (39%) and one soya-based formula (7%). Mg concentrations exceeded the maximum level in two samples of extensively hydrolyzed formulae, four samples of amino acid-based formulae, two samples of formulae manufactured from goat milk proteins, one sample of comfort formula, and 1 sample of anti-reflux formulae. Cu concentrations were below the minimum level in 26 samples, mainly in hydrolyzed formulae and exceeded the maximum in six samples (maximum 47%).

In summary, the concentrations of elements, except for Cu and K, were satisfactory for most formulae analyzed. Of the 149 formulae analyzed, the Cu concentrations were below the minimum in 43 samples and exceeded the maximum in 18 samples, whereas K concentrations exceeded the maximum in 63 samples.

### Estimated Daily Intake of the Trace Elements

The EDI of the trace elements is shown in Tables 4, 5, 6, 7, and 8. The EDIs for infant formulae from 1 to 6 months were varied and amounted to Na 96–355 mg/day, K 510–1325 mg/day, Ca 227–721 mg/day, P 183–543 mg/day, Mg 25–100 mg/day, Fe 1.2–13.8 mg/day, Zn 1.8–8.0 mg/day, Cu 0.0–1.8 mg/day, and Mn 2–290 µg/day (Table 4).

**Table 4:** Calculated daily infant intake of analyzed elements through consumption of infant formulae

Analyzed elements	Adequate intake for infants 0–6 months	Infant formulae (*n* = 37)		
		1 month	3 month	6 month
		Median (1st–3rd) < min–max >		
Na (mg/day)	120	168 (142–187) < 96–259 >	213 (180–238) < 121–328 >	230 (194–257) < 131–355 >
K (mg/day)	400	725 (635–801) < 510–967 >	920 (805–1016) < 647–1226 >	994 (871–1098) < 699–1325 >
Ca (mg/day)	200	391 (336–475) < 227–526 >	496 (426–602) < 287–667 >	536 (461–652) < 311–721 >
P (mg/day)	150	278 (233–321) < 183–397 >	353 (282–406) < 232–503 >	381 (305–439) < 251–543 >
Mg (mg/day)	30	53 (43–59) < 25–73 >	67 (54–74) < 32–93 >	72 (58–80) < 34–100 >
Fe (mg/day)	0.3	2.8 (2.3–3.7) < 1.2–10.1 >	3.6 (2.9–4.6) < 1.5–12.8 >	3.9 (3.1–5.0) < 1.6–13.8 >
Zn (mg/day)	2	3.2 (2.7–3.6) < 1.8–5.9 >	4.0 (3.5–4.5) < 2.3–7.4 >	4.3 (3.7–4.9) < 2.4–8.0 >
Cu (mg/day)	0.2	0.3 (0.2–0.4) < 0.0–1.3 >	0.4 (0.3–0.5) < 0.0–1.6 >	0.4 (0.3–0.5) < 0.0–1.8 >
Mn (µg/day)	3	42 (27–95) < 2–211 >	53 (34–121) < 2–268 >	58 (37–131) < 3–290 >

Obtained EDI values for Cu and Zn were compared to the Tolerable Upper Level (UL) for children aged 1 to 3 years. For Cu and Zn, the values were assumed to be 1 mg/day and 7 mg/day, respectively

**Table 5:** Calculated daily infant intake of analyzed elements through consumption of follow-on formulae

Analyzed elements	Adequate intake for infants 7–12 months*	Follow-on formulae (*n* = 34)	
		**7 months**	**10 months**
		**45% of the daily energy coverage**	**25% of the daily energy coverage**
		Median (1st–3rd) < min–max >	
Na (mg/day)	370	108 (87–117) < 67–175 >	68 (55–73) < 42–110 >
K (mg/day)	750	452 (389–491) < 293–826 >	283 (244–308) < 184–518 >
Ca (mg/day)	260	335 (292–368) < 179–510 >	210 (183–231) < 113–320 >
P (mg/day)	300	220 (195–240) < 87–239 >	138 (123–150) < 87–239 >
Mg (mg/day)	70	36 (27–42) < 11–73 >	23 (17–27) < 11–73 >
Fe (mg/day)	7**	2.9 (2.3–3.5) < 0.9–5.2 >	1.8 (1.4–2.2) < 0.6–3.2 >
Zn (mg/day)	2.5**	2.1 (1.9–2.8) < 1.6–4.0 >	1.3 (1.2–1.7) < 1.0–2.5 >
Cu (mg/day)	0.3	0.2 (0.2–0.3) < 0.0–**1.0** >	0.2 (0.1–0.2) < 0.0–0.6 >
Mn (µg/day)	600	49 (25–92) < 5–157 >	31 (16–58) < 3–98 >

*Norm regarding the consumption of all products throughout the day

**Norm in recommended daily intake

Obtained EDI values for Cu and Zn were compared to the Tolerable Upper Level (UL) for children aged 1 to 3 years. For Cu and Zn, the values were assumed to be 1 mg/day and 7 mg/day, respectively

**Table 6:** Calculated daily infant intake of analyzed elements through consumption of formula for special medical purposes intended for infants from birth to 6 months of age

**Analyzed elements**	**Adequate intake for infants** **0–6 months**	**Extensively hydrolyzed formulae** **(*n* = 14)**			**Formulae manufactured from goats’ milk proteins (*n* = 8)**			**Comfort-formulae** **(*n* = 3)**			**Anti-reflux formulae** **(*n* = 5)**			**Preterm formulae** **(*n* = 4)**		
		**1 month**	**3 months**	**6 months**	**1 month**	**3 months**	**6 months**	**1 month**	**3 months**	**6 months**	**1 month**	**3 months**	**6 months**	**1 month**	**3 months**	**6 months**
		Median (1st–3rd quartile) < min–max >														
Na (mg/day)	120	172	218	236	184	233	252	175	222	240	191	243	262	229	291	314
		(169–202)	(214–256)	(232–277)	(146–216)	(185–274)	(200–296)	(133–247)	(169–314)	(183–339)	(147–226)	(186–286)	(201–309)	(154–378)	(195–480)	(211–519)
		< 126–284 >	< 159–389 >	< 172–389 >	< 111–344 >	< 141–436 >	< 153–471 >	< 133–247 >	< 169–314 >	< 183–339 >	< 137–254 >	< 173–322 >	< 187–248 >	< 134–472 >	< 170–598 >	< 183–646 >
K (mg/day)	400	698	885	956	770	976	1055	622	789	853	667	845	913	648	822	889
		(666–801)	(844–1016)	(913–1098)	(627–884)	(9795–1121)	(859–1212)	(592–839)	(750–1064)	(811–1149	(615–765)	(780–971)	(843–1143)	(571–752)	(724–953)	(724–1030)
		< 624–909 >	< 792–1153 >	< 856–1246 >	< 513–1448 >	< 703–1985 >	< 703–1985 >	< 592–839 >	< 750–1064 >	< 811–1149 >	< 605–813 >	< 767–1031 >	< 829–1115 >	< 514–835 >	< 652–1058 >	< 704–1144 >
Ca (mg/day)	200	460	584	631	478	606	655	441	559	605	556	705	762	645.00	818	884
		(372–590)	(471–748)	(509–809)	(367–520)	(465–659)	(503–777)	(334–465)	(424–589)	(458–637)	(468–647)	(594–821)	(642–887)	(526–775)	(667–982)	(721–1062)
		< 252–849 >	< 320–1077 >	< 345–1164 >	< 252–835 >	< 320–1059 >	< 377–1248 >	< 334–465)	< 424–589 >	< 458–637 >	< 422–679 >	< 535–861 >	< 578–930 >	< 463–849 >	< 587–1077 >	< 634–1164 >
P (mg/day)	150	232	295	318	346	439	474	222	282	305	311	395	427	383	486	525
		(191–303)	(242–384)	(262–415)	(313–369)	(397–468)	(429–505)	(189–255)	(240–323)	(260–349)	(293–362)	(372–459)	(402–497)	(341–420)	(432–532)	(467–575)
		< 169–426 >	< 214–541 >	< 231–584 >	< 249–637 >	< 316–807 >	< 341–872 >	< 189–255 >	< 240–323 >	< 260–349 >	< 265–441 >	< 336–560 >	< 363–605 >	< 319–436 >	< 404–553 >	< 436–598 >
Mg (mg/day)	30	45	57	62	57	73	79	50	64	69	54	68	74	60	77	83
		(40–58)	(50–73)	(54–79)	(42–67)	(54–86)	(58–92)	(42–52)	(53–66)	(57–71)	(52–59)	(66–75)	(72–81)	(51–63)	(65–80)	(70–87)
		< 34–65 >	< 43–82 >	< 47–89 >	< 34–107 >	< 43–136 >	< 47–147 >	< 42–52 >	< 53–66 >	< 57–71 >	< 44–64 >	< 56–81 >	< 60–88 >	< 44–63 >	< 56–80 >	< 61–87 >
Fe (mg/day)	0.3	3.1	3.9	4.3	3.5	4.5	4.8	3.9	4.9	5.3	3.4	4.3	4.6	5.8	7.4	8.0
		(2.5–4.5)	(3.2–5.8)	(3.4–6.2)	(3.1–5.2)	(3.9–6.6)	(4.2–7.2)	(3.1–5.0)	(3.9–6.4)	(4.2–6.9)	(2.4–4.3)	(3.1–5.5)	(3.3–5.9)	(4.4–6.9)	(5.6–8.7)	(6.1–9.4)
		< 2.0–6.6 >	< 2.6–8.4 >	< 2.8–9.1 >	< 2.0–7.3 >	< 2.6–9.2 >	< 2.8–10.0 >	< 3.1–5.0 >	< 3.9–6.4 >	< 4.2–6.9 >	< 1.7–5.1 >	< 2.1–6.5 >	< 2.3–7.0 >	< 3.8–7.2 >	< 4.8–9.2 >	< 5.2–9.9 >
Zn (mg/day)	2	3.9	5.0	5.4	3.4	4.4	4.7	3.7	4.7	5.1	3.7	4.7	5.1	5.5	7.0	7.5
		(3.5–4.0)	(4.4–5.1)	(4.7–5.5.)	(3.0–4.5)	(3.8–5.7)	(4.1–6.1)	(3.6–4.1)	(4.5–5.1)	(4.9–5.6)	(3.7–4.0)	(4.6–5.0)	(5.0–5.4)	(4.8–5.9)	(6.1–7.5)	(6.6–8.1)
		< 3.1–4.7 >	< 3.9–5.9 >	< 3.9–5.9 >	< 2.7–**8.3** >	< 3.4–**8.3** >	< 3.7–**9.0** >	< 3.6–4.1 >	< 4.5–5.1 >	< 4.9–5.6 >	< 2.8–5.6 >	< 3.5–7.1 >	< 3.8–7.6 >	< 4.5–5.9 >	< 5.7–**7.5** >	< 6.1–**8.1** >
Cu (µg/day)	0.2	0.37	0.48	0.51	0.26	0.33	0.36	0.42	0.54	0.58	0.36	0.45	0.49	0.32	0.41	0.44
		(0.31–0.42)	(0.39–0.53)	(0.42–0.58)	(0.10–0.37)	(0.13–0.47)	(0.14–0.51)	(0.33–0.47)	(0.41–0.60)	(0.45–0.65)	(0.29–0.36)	(0.37–0.46)	(0.40–0.50)	(0.29–0.36)	(0.37–0.46)	(0.40–0.49)
		< 0.19–0.63 >	< 0.24–0.79 >	< 0.25–0.86 >	< 0.00–0.85 >	< 0.00–**1.08** >	< 0.00–**1.17** >	< 0.33–0.47 >	< 0.41–0.60 >	< 0.45–0.65 >	< 0.25–0.53 >	< 0.31–0.67 >	< 0.34–0.72 >	< 0.28–0.37 >	< 0.35–0.47 >	< 0.35–0.47 >
Mn (µg/day)	3	52	66	71	72	92	99	50	63	69	157	200	216	56	71	77
		(33–115)	(41–146)	(45–158)	(37–152)	(47–192)	(51–208)	(14–62)	(18–79)	(19–86)	(59–316)	(74–400)	(80–433)	(39–75)	(49–95)	(53–103)
		< 12–358 >	< 15–454 >	< 16–490 >	< **2**–211 >	< 3–268 >	< 3–289 >	< 14–62 >	< 18–79 >	< 19–86 >	< 17–347 >	< 22–439 >	< 23–475 >	< 36–80 >	< 45–101 >	< 49–110 >

Obtained EDI values for Cu and Zn were compared to the Tolerable Upper Level (UL) for children aged 1 to 3 years. For Cu and Zn, the values were assumed to be 1 mg/day and 7 mg/day, respectively

**Table 7:** Calculated daily infant intake of analyzed elements through consumption of formula for special medical purposes intended for infants from 6 to 12 months of age

Analyzed elements	Adequate intake for infants 7–12 months*	Extensively hydrolyzed formulae (*n* = 9)		Formulae manufactured from goats’ milk proteins (*n* = 7)		Comfort-formulae (*n* = 2)		Anti-reflux formulae (*n* = 1)		Soya-based formula for infants (*n* = 3)	
		**45% of the daily energy coverage**	**25% of the daily energy coverage**	**45% of the daily energy coverage**	**25% of the daily energy coverage**	**45% of the daily energy coverage**	**25% of the daily energy coverage**	**45% of the daily energy coverage**	**25% of the daily energy coverage**	**45% of the daily energy coverage**	**25% of the daily energy coverage**
		**7 months**	**10 months**	**7 months**	**10 months**	**7 months**	**10 months**	**7 months**	**10 months**	**7 months**	**10 months**
		Median (1st–3rd quartile) < min–max >									
Na (mg/day)	370	101	64	90	57	95	59	133	83	95	59
		(98–110) < 90–129 >	(61–69) < 56–81 >	(80–118) < 75–133 >	(50–74) < 47–84 >	(89–101) < 89–101 >	(56–63) < 56–63 >	– –	– –	(87–143) < 87–143 >	(55–90) < 55–90 >
K (mg/day)	750	490	308	477	299	363	228	495	310	380	238
		(410–522) < 355–592 >	(257–328) < 223–371 >	(371–545) < 315–569 >	(233–342) < 198–357 >	(340–386) < 340–386 >	(213–242) < 213–242 >	– –	– –	(369–450) < 369–450 >	(231–282) < 231–282 >
Ca (mg/day)	260	371	233	274	172	212	133	364	228	270	169
		(312–386) < 243–476 >	(196–242) < 152–299 >	(233–321) < 154–330 >	(149–202) < 97–207 >	(168–256) < 168–256 >	(106–161) < 106–161 >	– –	– –	(264–275) < 264–275 >	(166–172) < 166–172 >
P (mg/day)	300	215	135	216	135	154	97	282	177	293	184
		(178–230) < 159–250 >	(111–144) < 100–157 >	(166–236) < 150–248 >	(104–148) < 94–156 >	(138–171) < 138–171 >	(86–107) < 86–107 >	– –	– –	(185–305) < 185–305 >	(116–191) < 116–191 >
Mg (mg/day)	70	37	23	32	20	30	19	37	23	35	22
		(35–42) < 25–47 >	(22–26) < 16–30 >	(25–36) < 24–38 >	(16–23) < 15–24 >	(27–32) < 27–32 >	(17–20) < 17–20 >	– –	– –	(34–37) < 34–37 >	(21–23) < 21–23 >
Fe (mg/day)	7**	3.0	1.9	2.2	1.4	2.2	1.4	2.9	1.8	2.4	1.5
		(1.8–2.0) < 2.8–4.0 >	(1.8–2.0) < 1.7–2.5 >	(1.7–3.1) < 1.7–3.8 >	(1.1–2.0) < 1.0–2.4 >	(1.7–2.7) < 1.7–2.7 >	(1.1–1.7) < 1.1–1.7 >	– –	– –	(1.8–2.6) < 1.8–2.6 >	(1.2–1.6) < 1.2–1.6 >
Zn (mg/day)	2.5**	2.2	1.4	2.1	1.3	2.1	1.3	2.7	1.7	2.2	1.4
		(2.0–2.7) < 1.7–3.0 >	(1.3–1.7) < 1.1–1.9 >	(1.9–2.4) < 1.9–2.5 >	(1.2–1.5) < 1.2–1.5 >	(2.0–2.3) < 2.0–2.3 >	(1.2–1.4) < 1.2–1.4 >	– –	– –	(2.2–3.0) < 2.2–3.0 >	(1.4–1.9) < 1.4–1.9 >
Cu (µg/day)	0.3	0.16	0.10	0.17	0.10	0.19	0.12	0.44	0.27	0.19	0.12
		(0.15–0.26) < 0.14–0.29 >	(0.09–0.16) < 0.09–0.18 >	(0.12–0.17) < 0.08–0.22 >	(0.08–0.11) < 0.05–0.14 >	(0.14–0.24) < 0.14–0.24 >	(0.09–0.15) < 0.09–0.15 >	– –	– –	(0.12–0.21) < 0.12–0.21 >	(0.08–0.13) < 0.08–0.13 >
Mn (µg/day)	600	39	24	56	35	41	26	70	44	158	99
		(32–55) < 27–192 >	(20–34) < 17–121 >	(30–64) < 0–69 >	(19–40) < 0–43 >	(29–54) < 29–54 >	(18–34) < 18–34 >	– –	– –	(129–180) < 129–180 >	(81–113) < 81–113 >

*Norm regarding the consumption of all products throughout the day

**Norm in recommended daily intake

Obtained EDI values for Cu and Zn were compared to the Tolerable Upper Level (UL) for children aged 1 to 3 years. For Cu and Zn, the values were assumed to be 1 mg/day and 7 mg/day, respectively

**Table 8:** Calculated daily infant intake of analyzed elements through consumption of formula for special medical purposes intended for infants from birth to 12 months of age

Analyzed elements	Adequate intake for infants 0–6 months/ 7–12 months*	Extensively hydrolyzed formulae (*n*=3)				Amino acid-based (*n=6)*				Comfort formulae (*n=*7)			
		100% of the daily energy coverage		45% of the daily energy coverage	25% of the daily energy coverage	100% of the daily energy coverage		45% of the daily energy coverage	25% of the daily energy coverage	100% of the daily energy coverage		45% of the daily energy coverage	25% of the daily energy coverage
		1 month	6 months	7 months	10 months	1 month	6 months	7 months	10 months	1 month	6 months	7 months	10 months
		Median (1st – 3rd quartile) < min–max >											
Na (mg/day)	120/370	124	170	77	48	236	324	147	92	178	243	111	69
		(106–145)	(145–199)	(66–90)	(41–57)	(209–247)	(286–339)	(130–154)	(81–96)	(154–203)	(211–279)	(96–127)	(60–79)
		< 106–145>	< 145–199 >	< 66–90 >	< 41–57 >	< 203–269 >	< 278–369 >	< 126–168 >	< 79–105 >	< 149–232 >	< 204–318 >	< 93–145 >	< 58–91 >
K(mg/day)	400/750	587	804	365	229	735	1007	458	287	661	906	412	258
		(573–631)	(786–865)	(357–393)	(224–247)	(628–777)	(861–1064)	(391–484)	(245–303)	(579–722)	(794–989)	(361–449)	(226–282)
		< 573–631 >	< 786–865 >	< 357–393 >	< 224–247 >	< 521–900 >	< 713–1234 >	< 324–560 >	< 203–352 >	< 544–825 >	< 746–1131 >	< 339–514 >	< 213–322 >
Ca (mg/day)	200/260	419	574	217	164	520	712	324	203	426	443	265	166
		(349–455)	(479–623)	(217–283)	(136–178)	(490–541)	(672–742)	(305–337)	(192–211)	(409–574)	(560–787)	(254–357)	(160–224)
		< 349–455 >	< 479–623 >	< 217–283 >	< 136–178 >	< 460–613 >	< 631–841 >	< 287–382 >	< 180–240 >	< 323–583 >	< 443–799 >	< 201–363 >	< 126–228 >
P (mg/day)	150/300	245	336	153	96	355	486	221	139	308	422	192	120
		(214–393)	(294–538)	(133–245)	(84–153)	(274–408)	(376–558)	(171–254)	(107–159)	(304–333)	(417–456)	(189–207)	(119–130)
		< 214–393 >	< 294–538 >	< 133–245 >	< 84–153 >	< 248–450 >	< 340–616 >	< 154–280 >	< 97–176 >	< 191–379 >	< 262–520 >	< 119–236 >	< 75–148 >
Mg (mg/day)	30/70	41	56	25	16	74	101	46	29	58	79	36	22
		(34–45)	(47–62)	(21–28)	(13–18)	(55–76)	(79–104)	(36–47)	(22–30)	(47–68)	(64–94)	(29–42)	(18–27)
		< 34–45 >	< 47–62 >	< 21–28 >	< 13–18 >	< 44–76 >	< 61–105 >	< 28–47 >	< 17–30 >	< 43–71 >	< 60–98 >	< 27–44 >	< 17–28 >
Fe (mg/day)	0.3/7**	3.7	5	2.3	1.4	5.8	8	3.6	2.3	3.8	5.1	2.3	1.5
		(2.7–3.9)	(3.8–5.4)	(1.7–2.4)	(1.1–1.5)	(5.4–6.8)	(7.4–9.3)	(3.4–4.2)	(2.1–2.6)	(3.4–4.0)	(4.6–5.5)	(2.1–2.5)	(1.3–1.6)
		< 2.7–3.9 >	< 3.8–5.4 >	< 1.7–02.4 >	< 1.1–1.5 >	< 2.4–7.8 >	< 3.3–10.7 >	< 1.5–4.9 >	< 0.9–3.1 >	< 3.3–4.7 >	< 4.5–6.5 >	< 2.0–3.0 >	< 1.3–1.9 >
Zn (mg/day)	2/2.5**	3.5	4.8	2.2	1.4	4.1	5.7	2.6	1.6	4.4	6.1	2.8	1.7
		(3.4–4.3)	(4.6–5.8)	(2.1–2.7)	(1.3–1.7)	(4.1–5.0)	(5.6–6.9)	(2.6–3.1)	(1.6–2.0)	(3.6–4.6)	(4.9–6.3)	(2.2–2.9)	(1.4–1.8)
		< 3.4–4.3 >	< 4.6–5.8 >	< 2.1–2.7 >	< 1.3–1.7 >	< 3.6–5.1 >	< 5.0–7.0 >	< 2.3–3.2 >	< 1.4–2.0 >	< 2.8–4.6 >	< 3.8–6.3 >	< 1.7–2.9 >	< 1.1–1.8 >
Cu (µg/day)	0.2/0.3	0.4	0.5	0.2	0.1	0.4	0.6	0.3	0.2	0.2	0.4	0.2	0.1
		(0.2–0.4)	(0.3–0.6)	(0.1–0.3)	(0.1–0.2)	(0.3–0.6)	(0.3–0.9)	(0.2–0.4)	(0.1–0.3)	(0.2–0.3)	(0.3–0.5)	(0.3–0.2)	(0.1–0.1)
		< 0.2–0.4 >	< 0.3–0.6 >	< 0.1–0.3 >	< 0.1–0.2 >	< 0.4–0.7 >	< 0.3–1.0 >	< 0.2–0.5 >	< 0.1–0.3 >	< 0.2–0.8 >	< 0.2–1.1 >	< 0.1–0.5 >	< 0.1–0.3 >
Mn (µg/day)	3/600	254	347	158	99	147	202	92	58	275	377	171	107
		(12–320)	(17–439)	(18–199)	(5–125)	(89–193)	(121–264)	(55–120)	(35–75)	(110–301)	(151–413)	(69–187)	(43–118)
		< 12–320>	< 17–439 >	< 8–199 >	< 5–125 >	< 24–301>	< 33–413 >	< 15–188 >	< 10–118 >	< 71–419>	< 98–575 >	< 44–261 >	< 28–164 >

Analyzed elements	Anti-reflux formulae *(n*= 1)				Lactose-free formulae *(n* = 5)			
	100% of the daily energy coverage		45% of the daily energy coverage	25% of the daily energy coverage	100% of the daily energy coverage		45% of the daily energy coverage	25% of the daily energy coverage
	1 month	6 months	7 months	10 months	1 month	6 months	7 months	10 months
		Median (1 st – 3rd quartile) < min–max >						
Na (mg/day)	178	244	111	69	175	240	109	68
	–	–	–	–	(153–210)	(210–288)	(95–131)	(60–82)
	–	–	–	–	< 113–217>	< 155–298 >	< 70–135>	< 44–85>
K(mg/day)	739	1012	460	288	703	964	438	275
	–	–	–	–	(672–750)	(921–1028)	(418–467)	(262–293)
	–	–	–	–	< 565–897 >	< 774–1230 >	< 352–559 >	< 221–350 >
Ca (mg/day)	485	664	302	189	538	737	335	210
	–	–	–	–	(394–558)	(540–764)	(245–347)	(154–218)
	–	–	–	–	< 314–560 >	< 431–768 >	< 196–349 >	< 123–219 >
P (mg/day)	267	366	166	104	360	494	224	141
	–	–	–	–	(341–382)	(468–524)	(213–238)	(133–149)
	–	–	–	–	< 267–460 >	< 365–630 >	< 166–286 >	< 104–180 >
Mg (mg/day)	74	101	46	29	57	78	35	22
					(47–61)	(64–84)	(29–38)	(18–24)
					< 35–65 >	< 49–89 >	< 22–41 >	< 14–25 >
Fe (mg/day)	5.9	8.1	3.7	2.3	3.5	4.8	2.2	1.4
	–	–	–	–	(3.4–4.3)	(4.7–5.9)	(2.1–2.7)	(1.3–1.7)
	–	–	–	–	< 3.2–5.0 >	< 4.4–6.8 >	< 2.0–3.1 >	< 1.3–1.9 >
Zn (mg/day)	5.3	**7.2**	3.3	2.1	4.2	5.7	2.6	1.6
	–	–	–	–	(3.5–4.2)	(4.7–5.8)	(2.2–2.6)	(1.4–1.6)
	–	–	–	–	< 2.6–4.7 >	< 3.5–6.5 >	< 1.6–2.9 >	< 1.0–1.9 >
Cu (µg/day)	0.3	0.4	0.2	0.1	0.3	0.4	0.2	0.1
	–	–	–	–	(0.2–0.4)	(0.3–0.5)	(0.1–0.2)	(0.1–0.2)
	–	–	–	–	< 0.2–0.8 >	< 0.2–1.0 >	< 0.1–0.5 >	< 0.1–0.3 >
Mn (µg/day)	79	109	49	31	110	151	69	43
	–	–	–	–	(105–127)	(144–174)	(65–79)	(41–50)
	–	–	–	–	< 43–353 >	< 59–483 >	< 27–220 >	<17–138>

*Norm regarding the consumption of all products throughout the day

**Norm in recommended day intake

Obtained EDI values for Cu and Zn were compared to the Tolerable Upper Level (UL) for children aged 1 to 3 years. For Cu and Zn, the values were assumed to be 1 mg/day and 7 mg/day, respectively.

 The EDIs for infants in the 1 st month of life were lower than the AI for Na in three formulae, Zn in one formula, and Mg in one sample, but the Mg concentrations met minimum levels per EU guidelines. The EDI values for Mn were below AI for infants at 1 and 3 months of life for one formula, despite it meeting the EU criteria for minimum levels. The EDIs below AI for Zn and Cu were in those formulae with concentrations below the minimum levels specified by the EU (Table 1).

Regarding the EDIs to UL for Zn and Cu, it was observed that some formulae estimated intake was too high, especially for infants at 3 and 6 months of age for Zn (in one formula), and for infants at 1, 3, and 6 months of age for Cu (in two formulae) but it exceeded the maximum levels.

For follow-on formulae, the EDI was assessed in infants aged 7 and 10 months, taking into account the energy obtained from the formulae as 45% and 25% of the total daily requirement, respectively (Table 5). The median EDI for follow-on formulae for 7-month infants was 108 mg/day for Na, representing 29% of the AI, 452 mg/day for K (60% of AI), 335 mg/day for Ca (129% of AI), 220 mg/day for P (73% of AI), 36 mg/day for Mg (51% of AI), 2.9 mg/day for Fe (41% of RDA), 2.1 mg/day for Zn (84% of RDA), 0.2 mg/day for Cu (67% of AI), and 49 µg/day for Mn (8% of AI). Although when calculating the EDI for infants aged 7 months, we assumed that the energy requirement from milk was covered by only 45% of the energy value of the diet, the EDI for Cu exceeded the UL values in one analyzed formula.

The formulae for special medical purposes were divided into three groups according to the manufacturer’s declaration regarding the age range of the intended infants: infants 0–6 months (Table 6), infants aged 7–12 months (Table 7), and infants 0–12 months (Table 8). An analysis of EDIs of trace elements showed that most formulae intended for 0–6 months met nutrition recommendations, except for Mn, Zn, and Cu. The EDI for Mn was below AI for infants at 1 month in one goat formula despite meeting the EU criteria for minimum level (Table 6). The EDI for Zn and Cu exceeded UL for four formulae (two goat and two preterm formulae) and exceeded the maximum values allowed by the EU.

Table 7 presents the EDIs for formulae for special medical purposes intended for infants aged 6 to 12 months, showing that no formulae exceeded the UL, so consumption of the formula in combination with a complementary diet should fully cover all nutrient requirements. However, the average EDI for Fe for infants aged 7 months (results only for formula consumption) was 2.5 mg, 36% of the AI; therefore, foods rich in Fe should be consumed by infants starting to expand their diet.

Table 8 presents the EDIs for formulae for special medical purposes intended for infants aged 0 to 12 months, showing that they met the dietary recommendations for most elements except Na, with one extensively hydrolyzed formula and one lactose-free formula below the AI. Comparing EDIs to UL values, Zn and Cu were exceeded in some formulae, Cu in three formulae, and Zn in two formulae.

### Toxic and Potentially Toxic Elements

The Cd, Hg, As, Sn, Co, Cr, and Ni concentrations are presented in Table 9. The concentration was converted to µg/kg mass formulae to compare maximum levels for specific contaminants in food and repealing Regulation (UE) 2023/915 of April 25, 2023/Commission Regulation (EC) No. 1881/2006 of December 19, 2006 [20]. Cd, Hg, and As concentrations were below the detection limit in all formulae. Co concentrations in most formulae were below the limit detection level, but the remaining formulae did not exceed 10 µg/kg wet weight (maximum level). The average Sn and Cr concentrations were low and amounted to 0.3 µg/kg and 0.0 µg/kg wet weight, respectively. The Ni concentrations varied, but most were below the detection limit. This metal was detected in 62 of 149 samples, with the highest concentration of 17 µg/kg wet–weight powder formulae.

**Table 9:** Toxic and potential toxic elements (µg/kg wet weight) in all analyzed formulae

**Analyzed elements**	**Limits** (µg/kg)	**Infant formulae** **(0–6 months)** **(*n* = 37)**	**Follow-on formulae** **(7–12 months)** **(*n* = 34)**	**Formula for special medical purposes intended for infants** **(*n* = 78)**
		Median (1st–3rd quartile) < min–max >		
Cd (µg/kg)	10	< DL	< DL	< DL
Hg (µg/kg)	–	< DL	< DL	< DL
As (µg/kg)	20	< DL	< DL	< DL
Sn (µg/kg)	50 000	0.3 (0.1–0.5) < 0–2.2 >	0.2 (0.2–0.4) < 0–1.1 >	0.4 (0.2–0.6) < 0–11 >
Co (µg/kg)	–	0 (0–0) < 0–1.0 >	0 (0–0) < 0–1.1 >	0 (0–0) < 0–9.6 >
Cr (µg/kg)	–	0 (0–0) < 0–23 >	0 (0–0) < 0–13 >	0 (0–0) < 0–1.7 >
Ni (µg/kg)	–	0 (0–0) < 0–17 >	0 (0–6.0) < 0–13 >	0 (0–6.5) < 0–12 >

Values of DL (µg/L): Cd: 0.45; Hg: 18; As: 6.0; Sn: 0.27; Co: 1.5; Cr: 0.77; Ni: 13

### Estimated Daily Intake of Toxic and Potentially Toxic Elements

The EDIs were calculated for Sn, Co, Cr, and Ni (Table 10) and the EDI per kilogram of body weight per day was calculated for Cr and Ni to compare the values with the recommendations.

**Table 10:** Calculated daily infant intake of analyzed elements through consumption of analyzed formulae

Analyzed elements	Infant formulae (0–6 months) (*n* = 37)			Follow-on formulae (7–12 months) (*n* = 34)		Formula for special medical purposes intended for infants (*n* = 78)			
	Median (1st–3rd quartile) < min–max >								
	1 month	3 months	6 months	7 months	**10 months**	**1 month**	**6 months**	**7 months**	**10 months**
	**Calculated for 100% of the daily energy coverage**			**45% of the daily energy coverage**	**25% of the daily energy coverage**		**Calculated for 100% of the daily energy coverage**	**45% of the daily energy coverage**	**25% of the daily energy coverage**
Sn (µg/day)	1.86 (0.00–3.31) < 0.00–15.66 >	2.37 (0.00–4.20) < 0.00–19.86 >	2.56 (0.00–4.54) < 0.00–21.47 >	0.91 (0.68–1.54) < 0.00–4.70 >	0.57 (0.43–0.97) < 0.00–2.95 >	2.42 (1.14–3.89) < 0.00–76.60 >	3.31 (1.56–5.33) < 0.00–104.97 >	1.51 (0.71–2.42) < 0.00–47.69 >	0.94 (0.44–1.52) < 0.00–29.91 >
Co (µg/day)	0.00 (0.00–0.00) < 0.00–6.77 >	0.00 (0.00–0.00) < 0.00–8.58 >	0.00 (0.00–0.00) < 0.00–9.27 >	0.00 (0.00–0.00) < 0.00–4.52 >	0.00 (0.00–0.00) < 0.00–2.84 >	0.00 (0.00–0.00) < 0.00–64.49 >	0.00 (0.00–0.00) < 0.00–88.38 >	0.00 (0.00–0.00) < 0.00–40.15 >	0.00 (0.00–0.00) < 0.00–25.19 >
Cr (µg/day)	0.00 (0.00–0.00) < 0.00–153.53 >	0.00 (0.00–0.00) < 0.00–194.68 >	0.00 (0.00–0.00) < 0.00–210.40 >	0.00 (0.00–0.00) < 0.00–53.68 >	0.00 (0.00–0.00) < 0.00–33.68 >	0.00 (0.00–0.00) < 0.00–12.04 >	0.00 (0.00–0.00) < 0.00–16.50 >	0.00 (0.00–0.00) < 0.00–7.49 >	0.00 (0.00–0.00) < 0.00–4.70 >
^*^Cr (mg/day/ kg body weight]	0.00 (0.00–0.00) < 0.00–0.02 >	0.00 (0.00–0.00) < 0.00–0.03 >	0.00 (0.00–0.00) < 0.00–0.03 >	0.00 (0.00–0.00) < 0.00–0.01 >	0.00 (0.00–0.00) < 0.00–0.00 >	0.00 (0.00–0.00) < 0.00–0.00 >	0.00 (0.00–0.00) < 0.00–0.00 >	0.00 (0.00–0.00) < 0.00–0.00 >	0.00 (0.00–0.00) < 0.00–0.00 >
Ni (µg/day)	0.00 (0.00–0.00) < 0.00–115.29 >	0.00 (0.00–0.00) < 0.00–146.18 >	0.00 (0.00–0.00) < 0.00–157.99 >	0.00 (0.00–25.42) < 0.00–56.13 >	0.00 (0.00–15.95) < 0.00–35.21 >	0.00 (0.00–44.40) < 0.00–78.44 >	0.00 (0.00–60.85) < 0.00–107.50 >	0.00 (0.00–27.64) < 0.00–48.83 >	0.00 (0.00–17.34) < 0.00–30.63 >
^**^Ni (µg/day/ kg body weight)	0.00 (0.00–0.00) < 0.00–**18.36** >	0.00 (0.00–0.00) < 0.00–**19.08** >	0.00 (0.00–0.00) < 0.00–**19.29** >	0.00 (0.00–3.08) < 0.00–**6.80** >	0.00 (0.00–1.74) < 0.00–3.84 >	0.00 (0.00–**7.07**) < 0.00–**12.49** >	0.00 (0.00–**7.43**) < 0.00–**13.13** >	0.00 (0.00–3.35) < 0.00–**5.91** >	0.00 (0.00–1.88) < 0.00–3.34 >

*Obtained EDI values for Cr compared to estimated safe and adequate daily dietary intake – 0.01–0.04 mg/day, and adequate Cr intake levels lie between 0.0001 and 0.001 mg/body weight/day. **For Ni to tolerable daily intake – 13 µg/kg body weight, and the lowest observed adverse effect level for eczematous flare–up reactions in the skin elicited – 4.3 μg/kg body weight

The EDI for Sn was varied (0.00–104.95 µg/day) with a maximum value of 105 µg/day for the formula for special medical purposes for infants ages 6 months. The EDI for Sn for infant formulae and follow-on formulae was lower than formulae for special medical purposes.

Co was not detected in most preparations, with a maximum EDI in infant formulae for 1 month (6.77 µg/day) and 6 months (9.27 µg/day). Regarding the follow-on formula, the maximum EDI was lower (4.52 and 2.95 µg/day calculated for 75% of the daily energy coverage for infants in 7 months and 45% for infants in 10 months). For formulae for special medical purposes, the EDI was much higher (64.49 for infants in 1 month of life and 6 months of life).

The EDI for Cr was varied (0.00–210.40 µg/day) with the highest median EDIs for infant formulae and the lowest for formulae for special medical purposes. Additionally, the EDI per kg body weight was estimated. According to the National Research Council, the estimated safe and adequate daily dietary intake of Cr is 0.01–0.04 mg/day for infants [23] and none of the analyzed formulae exceeded these values. According to the Committee on Medical Aspects of Food and Nutrition Policy, an adequate level of chromium intake is between 0.0001 and 0.001 mg/body weight/day for children.

The EDI for Ni was varied (0.00–157.99 µg/day). The EDI was converted to µg/kg body weight for comparison to the TDI for Ni of 13 µg/kg body weight, and the LOAEL for eczematous flare-up reactions of 4.3 μg/kg body weight [21]. The EDI in four formulae for infants aged 6 months and two formulae for infants aged 1 month exceeded the TDI. The EDI in all formulae, regardless of infant formulae or formula for special medical purposes, from 1 to 6 months of life, exceeded the LOAEL (4.3 µg) established by EFSA.

Obtained EDI values for Cr compared to estimated safe and adequate daily dietary intake—0.01–0.04 mg/day, and adequate Cr intake levels lie between 0.0001 and 0.001 mg/body weight/day. For Ni to tolerable daily intake—13 µg/kg body weight, and the lowest observed adverse effect level for eczematous flare-up reactions in the skin elicited—4.3 μg/kg body weight.

## Discussion

The composition of formulae for infants is constantly changing, and the consumption of modified formulae as an alternative to breast milk is high, so the element concentrations should be regularly analyzed.

Nutrition in the first year of life plays a key role in a child’s development. According to WHO recommendations, the daily energy requirement from birth to 6 months of age should be supplied by the mother’s milk or, if not possible, infant formula. However, breast milk or infant formula cannot cover all the required nutrients when infants start to consume different foods to expand their diet [24]. Piccinelli’s study showed that in the seventh month of life, infant formula should only supply 45% of energy requirements and 25% at 10 months.

In most of the formulae for infants analyzed in this study, the element concentrations were within the EU guidelines, except for Cu and K. Moreover, formulae that did not meet the EU guidelines also did not meet the nutritional guidelines, with Cu EDIs below or above the permissible levels and an AI deficiency or a UL excess. Interestingly, regarding elements such as Na and Mn, despite meeting EU minimum and maximum limits, some formulae did not meet the AI. In particular, the Mn EDIs for infants in the first month of life were 2 µg/day (67% of AI) and some at 120 µg/day above the AI. The EDI values for Zn were lower than the AI or exceeded the UL, which was related to the failure to meet the EU guidelines regarding maximum and minimum levels.

The Cu concentrations in the analyzed formulae were slightly higher than the average Cu content reported in formulae in Europe. The median Cu concentration in all analyzed formulae was 72 µg/100 kcal (66 µg/100 kcal for infant formulae, 81 µg/100 kcal for follow-on formulae, and 68 µg/100 kcal for special medical purposes formulae) and the mean Cu concentration in breast milk observed in Europe ranges from 51 to 60 µg/100 kcal. However, the large discrepancy between the Cu concentrations in the formulae is concerning. As previously mentioned, 71 formulae did not meet the guidelines regarding the content limits, which influenced the EDIs. The EDI would not exceed UL if the concentrations were within the recommended limits (60–100 µg/100 kcal for infant formulae and follow-on formulae or 60–120 µg/100 kcal for special medical purposes). Detailed analysis showed that UL was exceeded in nine different formulae, of which four formulae for infants in the first month of life exceeded UL values. Cu concentrations of 3.75 mg/kg have been reported, and the calculated average EDI for the tested formulae was 188% of the recommended daily intake [25]. Also, Cu concentrations have been reported to be significantly higher than the reference values, possibly due to the product packaging [9]. Previously, we reported Cu concentrations exceeding the limits in two formulae [26] but other studies found that Cu concentrations did not exceed recommended values [27].

The K concentrations in formulae for infants were similar to other reports [2, 28, 29]. In our research, the K EDI sometimes is twice the AI values, making monitoring the element concentrations in formulae necessary. Prolonged high K intake can lead to high concentrations of blood K that may affect cardiac function, especially in infants with impaired kidney function [18]. It should be emphasized, however, that for all formulae with reduced Na concentrations, the K concentration was proportionally lower, and each formula was within the norm.

The variations in Mn concentrations in the studied formulae will cause significant differences in Mn intake in infants. According to Mullee et al., the mean Mn concentration in human milk of European mothers varies from 0.46 to 4.6 µg/100 kcal [2, 30]. The EFSA concluded that a 3 µg/day Mn intake in the first half of the year of life was adequate for most infants, and in the second year of life, it was higher at 20–500 µg/day [2]. Mn is an essential component of metalloenzymes such as superoxide dismutase, arginase, and pyruvate carboxylase and is involved in amino acid, lipid, and carbohydrate metabolism. However, in large amounts, it may cause toxic effects and result in a permanent neurological disorder [31]. Symptoms consist of reduced response speed, irritability, mood changes, and compulsive behaviours [32]. Based on the available literature, only neurotoxic effects were reported by Fell et al. (1996) with whole blood Mn concentrations in the children ranging from 9.9 to 110 μg/L [33]. Other studies also indicate large differences in Mn concentrations between formulae and point out that the Mn intake may be excessive in some cases [31, 34–37]. Mitchell’s study showed the EDI Mn intake from formulae for infants ranged from 130 μg/kg/day (3 weeks) to 4.8 μg/kg/day (18 months) [35], whereas Frisbie et al. reported Mn concentrations in infant formulae of 160 to 2800 μg/L, with 7 of 25 products purchased in the US exceeding the upper level [38]. Moreover, the Mn concentrations of the water used to prepare the formula should also be taken into account, so the infants’ total Mn intake may be even higher. According to the WHO, a median drinking water level of 10 μg/L [39].

Concerning toxic and potentially toxic elements, the concentrations in the formulae were below the maximum levels and similar to other studies [8, 40, 41]. Only EDI values for Ni indicated that some formulae exceeded the TDI. Interestingly, in all the analyzed formulae (62 out of 149 samples) in which Ni was detected, the EDI for infants at 1 month exceeded the LOAEL, which may increase the risk of eczema in sensitive individuals. It should be mentioned that the total Ni concentrations in the formulae (powder and water) may be higher due to the Ni content of the water used to prepare the formulae [42]. Furthermore, some studies suggest that children should not take more than 100 µg/day of Ni considering their lower body weight [43].

Analyzing the EDI value for infants after 6 months of age, the period of expanding the diet shows that the estimated intake of Cu for some formulae is too high. Although the calculated EDI was only 45% of the daily energy from the formula, the value for 7-month-old infants in one follow-on formula exceeded the UL. The estimated consumption of the remaining analyzed elements was appropriate, except for Fe and Mn which were not sufficient. Since most formulae will not cover the demand (AI values), it is important to introduce food products rich in Fe and Mn during this period [44, 45]. It is of note that the main sources of Mn in the human diet are whole grains and cereals [27]. Interestingly, the estimated Ca intake from most milk formulae was sufficient (median close to the AI value). The analyzed EDI values for potentially toxic elements showed that the estimated Ni intake is too high. If products rich in Ni (chocolate and nuts) are eaten during this period, allergic symptoms may intensify in sensitive people, and the TDI value may be exceeded. Thus, it is recommended to control the concentration of this element in formulae and establish safety limits.

The assessment of Pb and Se concentrations is limited in this study due to significant elemental interference in Pb concentrations. Furthermore, the Se concentrations will appear in a separate publication [46].

## Conclusion

- The estimated Cu intake exceeded the upper level in some formulae, so regular monitoring of the Cu content is required. Future studies should consider whether this carries negative health consequences for infants.
- There were significant disproportions between Mn concentrations in formulae related to broad standards regarding minimum and maximum levels. Future studies should consider whether this carries negative health consequences for infants.
- Ni was detected in some formulae and its concentration exceeded the LOAEL standards, which may pose a risk of eczema in sensitive individuals. Additionally, its content exceeds the TDI in some cases, so it is recommended to introduce limits on its content in infant products.
- The best method of feeding infants up to 1 year of age is breastfeeding, but if this is not possible, a product adapted to the infant’s needs should be chosen. This study confirms that formulae must be adapted to the infant’s age and developmental needs as formulae that are inappropriate for the age range may cause an excess or deficiency of some elements. Additionally, when expanding the child’s diet, foods should be selected from all food groups, particularly those rich in iron, and limiting the consumption of products naturally rich in Ni.

Regular monitoring of the composition of infant formula is recommended. Modifications to EU guidelines regarding Mn limits and the introduction of guidelines regarding the Ni content in infant formulae should also be considered.

## Data Availability

No datasets were generated or analysed during the current study.

## References

[1] Froń A, Orczyk-Pawiłowicz M (2024) Breastfeeding beyond six months: evidence of child health benefits. Nutrients 16:3891. 10.3390/nu16223891 39599677 10.3390/nu16223891 PMC11597163

[2] Scientific Opinion on the essential composition of infant and follow-on formulae. EFSA Journal. 10.2903/j.efsa.2014.3760

[3] Breastfeeding [Internet]. (2022) Available from: UNICEF 2022 https://data.unicef.org/topic/nutrition/breastfeeding

[4] World Health Organization. Breastfeeding. (2024). Available from:https://www.who.int/news-room/facts-in-pictures/detail/breastfeeding

[5] Victora CG, Bahl R, Barros AJD, França GVA, Horton S, Krasevec J, Murch S, Sankar MJ, Walker N, Rollins NC (2016) Breastfeeding in the 21st century: epidemiology, mechanisms, and lifelong effect. Lancet 387:475–490. 10.1016/S0140-6736(15)01024-7 26869575 10.1016/S0140-6736(15)01024-7

[6] Global breastfeeding scorecard 2023 [Internet]. (2023) Available from: https://www.unicef.org/media/150586/file/Global%20breastfeeding%20scorecard%202023.pdf

[7] Michaelsen KF, Weaver L, Branca F, Robertson A (2003) Feeding and nutrition of infants and young children: guidelines for the WHO European Region, with emphasis on the former Soviet countries. World Health Organization, Regional Office for Europe. https://iris.who.int/handle/10665/272658

[8] Pandelova M, Lopez WL, Michalke B, Schramm K-W (2012) Ca, Cd, Cu, Fe, Hg, Mn, Ni, Pb, Se, and Zn contents in baby foods from the EU market: comparison of assessed infant intakes with the present safety limits for minerals and trace elements. J Food Compos Anal 27:120–127. 10.1016/j.jfca.2012.04.011

[9] Demir T, Ağaoğlu S (2023) Estimated daily intake and health risk assessment of toxic elements in infant formulas. Br J Nutr 130:1732–1742. 10.1017/S0007114523000971 37066728 10.1017/S0007114523000971

[10] Lin X, Wu X, Li X, Zhang D, Zheng Q, Xu J, Lu S (2022) Infant exposure to trace elements in breast milk, infant formulas and complementary foods from Southern China. Sci Total Environ 838:156597. 10.1016/j.scitotenv.2022.156597 35690194 10.1016/j.scitotenv.2022.156597

[11] Chajduk E, Pyszynska M, Polkowska-Motrenko H (2018) Determination of trace elements in infant formulas available on polish market. Biol Trace Elem Res 186:589–596. 10.1007/s12011-018-1339-5 29679351 10.1007/s12011-018-1339-5

[12] Purkiewicz A, Mumtaz W, Tońska E, Pietrzak-Fiećko R (2024) Mineral content in initial and follow-on infant formulas in Poland: nutrient adequacy and comparison with breast milk. Appl Sci 14:10235. 10.3390/app142210235

[13] Jardí Piñana C, Aranda Pons N, Bedmar Carretero C, Arija Val V (2015) Nutritional composition of infant milk formulas. Level of compliance in their manufacture and adequacy of nutritional needs. Anales de Pediatría (English Edition) 83:417–429. 10.1016/j.anpede.2015.10.006 10.1016/j.anpedi.2015.03.003 25869792

[14] Papachristodoulou C, Tsiamou M-C, Sakkas H, Papadopoulou C (2018) Determination of minerals in infant milk formulae by energy dispersive X-ray fluorescence spectrometry. J Food Compos Anal 72:39–47. 10.1016/j.jfca.2018.06.007

[15] Yaman M, Çokol N (2004) Determination of trace elements in human milk, cow’s milk, and baby foods by flame AAS using wet ashing and microwave oven sample digestion procedures. At Spectrosc 25:185–190

[16] COMMISSION DELEGATED REGULATION (EU) 2016/127 of 25 September 2015 supplementing Regulation (EU) No 609/2013 of the European parliament and of the council as regards the specific compositional and information requirements for infant formula and follow-on formula and as regards requirements on information relating to infant and young child feeding.

[17] COMMISSION DELEGATED REGULATION (EU) 2016/128 of 25 September 2015 supplementing Regulation (EU) No 609/2013 of the European Parliament and of the Council as regards the specific compositional and information requirements for food for special medical purposes.

[18] Stan SV, Grathwohl D, O’Neill LM, Saavedra JM, Butte NF, Cohen SS (2021) Estimated energy requirements of infants and young children up to 24 months of age. Current developments. Nutrition 5:nzab122. 10.1093/cdn/nzab122 10.1093/cdn/nzab122 PMC8575726 34761158

[19] Piccinelli R, Pandelova M, Le Donne C, Ferrari M, Schramm K-W, Leclercq C (2010) Design and preparation of market baskets of European Union commercial baby foods for the assessment of infant exposure to food chemicals and to their effects. Food Addit Contam A 27:1337–1351. 10.1080/19440049.2010.489913 10.1080/19440049.2010.489913 20635267

[20] COMMISSION REGULATION (EU) 2023/915 of 25 April 2023 on maximum levels for certain contaminants in food and repealing Regulation (EC) No 1881/2006

[21] EFSA Panel on Contaminants in the Food Chain (CONTAM), Schrenk D, Bignami M, Bodin L, Chipman JK, del Mazo J, Grasl‐Kraupp B, Hogstrand C, Hoogenboom L (Ron), Leblanc J, Nebbia CS, Ntzani E, Petersen A, Sand S, Schwerdtle T, Vleminckx C, Wallace H, Guérin T, Massanyi P, Van Loveren H, Baert K, Gergelova P, Nielsen E (2020) Update of the risk assessment of nickel in food and drinking water. EFS2 18. 10.2903/j.efsa.2020.6268 10.2903/j.efsa.2020.6268 PMC7643711 33193868

[22] Scientific opinion on dietary reference values for chromium. EFSA Journal. 10.2903/j.efsa.2014.3845 10.2903/j.efsa.2014.3845 PMC13136993 42087961

[23] Safe upper levels for vitamins and minerals. afe upper levels for vitamins and minerals. Food Standards Agency, 2003 https://cot.food.gov.uk/sites/default/files/vitmin2003.pdf

[24] Woźniak D, Podgórski T, Dobrzyńska M, Przysławski J, Drzymała S, Drzymała-Czyż S (2022) The influence of parents’ nutritional education program on their infants’ metabolic health. Nutrients 14:2671. 10.3390/nu14132671 35807852 10.3390/nu14132671 PMC9268789

[25] Paz S (2017) Essential and toxic metals in infant formula from the European community. OAJT 2. 10.19080/oajt.2017.02.555585

[26] Dobrzyńska M, Drzymała-Czyż S, Jakubowski K, Kurek S, Walkowiak J, Przysławski J (2021) Copper and zinc content in infant milk formulae available on the polish market and contribution to dietary intake. Nutrients 13:2542. 10.3390/nu13082542 34444702 10.3390/nu13082542 PMC8400833

[27] Peirovi-Minaee R, Taghavi M, Harimi M, Zarei A (2024) Trace elements in commercially available infant formulas in Iran: determination and estimation of health risks. Food Chem Toxicol 186:114588. 10.1016/j.fct.2024.114588 38467297 10.1016/j.fct.2024.114588

[28] Noble S, Emmett P (2006) Differences in weaning practice, food and nutrient intake between breast- and formula-fed 4-month-old infants in England. J Human Nutrition Diet 19:303–313. 10.1111/j.1365-277x.2006.00708.x 16911243 10.1111/j.1365-277X.2006.00708.x

[29] Lennox A, Sommerville J, Ong K, Henderson H and Allen R (2013). Diet and nutrition survey of infants and young children, 2011. A survey carried out on behalf of the Department of health and food standards agency. Available online: https://webarchive.nationalarchives.gov.uk/20130402145952/https://transparency.dh.gov.uk/2013/03/13/dnsiyc-2011/

[30] Mullee A, Brown T, Collings R, Harvey L, Hooper L and Fairweather-Tait S (2012) Literature search and review related to specific preparatory work in the establishment of dietary reference values. Preparation of an evidence report identifying health outcomes upon which dietary reference values could potentially be based for chromium, manganese and molybdenum European food safety authority 2012.

[31] Scher DP, Goeden HM, Klos KS (2021) Potential for manganese-induced neurologic harm to formula-fed infants: a risk assessment of total oral exposure. Environ Health Perspect 129:047011.10.1289/EHP7901 33848192 10.1289/EHP7901 PMC8043326

[32] ATSDR (Agency for Toxic Substances and Disease Registry). (2012) Toxicological profile for Manganese. U.S. Department of Health and Human Services, Washington DC, USA, p 556

[33] Fell JME, Meadows N, Khan K, Long SG, Milla PJ, Reynolds AP, Quaghebeur G, Taylor WJ (1996) Manganese toxicity in children receiving long-term parenteral nutrition. The Lancet 347:1218–1221. 10.1016/S0140-6736(96)90735-7 10.1016/s0140-6736(96)90735-7 8622451

[34] Mitchell EJ, Frisbie SH, Roudeau S, Carmona A, Ortega R (2021) How much manganese is safe for infants? A review of the scientific basis of intake guidelines and regulations relevant to the manganese content of infant formulas. J Trace Elem Med Biol 65:126710. 10.1016/j.jtemb.2020.126710 33450552 10.1016/j.jtemb.2020.126710

[35] Mitchell EJ, Frisbie SH, Roudeau S, Carmona A, Ortega R (2020) Estimating daily intakes of manganese due to breast milk, infant formulas, or young child nutritional beverages in the United States and France: Comparison to sufficiency and toxicity thresholds. J Trace Elem Med Biol 62:126607. 10.1016/j.jtemb.2020.126607 32683229 10.1016/j.jtemb.2020.126607

[36] Coetzee DJ, McGovern PM, Rao R, Harnack LJ, Georgieff MK, Stepanov I (2016) Measuring the impact of manganese exposure on children’s neurodevelopment: advances and research gaps in biomarker-based approaches. Environ Health 15:91. 10.1186/s12940-016-0174-4 27576472 10.1186/s12940-016-0174-4 PMC5004305

[37] Yoon M, Schroeter JD, Nong A, Taylor MD, Dorman DC, Andersen ME, Clewell HJ (2011) Physiologically based pharmacokinetic modeling of fetal and neonatal manganese exposure in humans: describing manganese homeostasis during development. Toxicol Sci 122:297–316. 10.1093/toxsci/kfr141 21622944 10.1093/toxsci/kfr141

[38] Frisbie SH, Mitchell EJ, Roudeau S, Domart F, Carmona A, Ortega R (2019) Manganese levels in infant formula and young child nutritional beverages in the United States and France: comparison to breast milk and regulations. PLoS ONE 14:e0223636. 10.1371/journal.pone.0223636 31689314 10.1371/journal.pone.0223636 PMC6830775

[39] WHO. Background document for development of WHO guidelines for drinking-water quality. World Health Organization; 2004b. Manganese in drinking-water. WHO/SDE/WSH/03.04/104. http://www.who.int/water_sanitation_health/dwq/chemicals/manganese.pdf. 07 April 2008.

[40] Dabeka RW (1989) Survey of lead, cadmium, cobalt and nickel in infant formulas and evaporated milks and estimation of dietary intakes of the elements by infants 0–12 months old. Sci Total Environ 89:279–289. 10.1016/0048-9697(89)90267-2 2617291 10.1016/0048-9697(89)90267-2

[41] Başaran B (2022) An assessment of heavy metal level in infant formula on the market in Turkey and the hazard index. J Food Compos Anal 105:104258. 10.1016/j.jfca.2021.104258

[42] Darsow U, Fedorov M, Schwegler U, Twardella D, Schaller K, Habernegg R, Fromme H, Ring J, Behrendt H (2012) Influence of dietary factors, age and nickel contact dermatitis on nickel excretion. Contact Dermatitis 67:351–358. 10.1111/j.1600-0536.2012.02153.x 22928956 10.1111/j.1600-0536.2012.02153.x

[43] Mislankar M, Zirwas MJ (2013) Low-nickel diet scoring system for systemic nickel allergy. Dermatitis 24:190–195. 10.1097/DER.0b013e3182937e81 23857013 10.1097/DER.0b013e3182937e81

[44] Woźniak D, Podgórski T, Krzyżanowska-Jankowska P, Dobrzyńska M, Wichłacz-Trojanowska N, Przysławski J, Drzymała-Czyż S (2022) The influence of intensive nutritional education on the iron status in infants. Nutrients 14:2453. 10.3390/nu14122453 35745183 10.3390/nu14122453 PMC9229227

[45] Woźniak D, Przysławski J, Banaszak M, Drzymała-Czyż S (2022) Dietary supplements among children ages 0–3 years in Poland—are they necessary? Foods 12:16. 10.3390/foods12010016 36613232 10.3390/foods12010016 PMC9818416

[46] Dobrzyńska M, Kaczmarek K, Przysławski J, Drzymała-Czyż S (2023) Selenium in infants and preschool children nutrition: a literature review. Nutrients 15:4668. 10.3390/nu15214668 37960322 10.3390/nu15214668 PMC10648445

